# Within-host evolution of *Enterococcus faecium* during longitudinal carriage and transition to bloodstream infection in immunocompromised patients

**DOI:** 10.1186/s13073-017-0507-0

**Published:** 2017-12-27

**Authors:** Danesh Moradigaravand, Theodore Gouliouris, Beth Blane, Plamena Naydenova, Catherine Ludden, Charles Crawley, Nicholas M. Brown, M. Estée Török, Julian Parkhill, Sharon J. Peacock

**Affiliations:** 1 Wellcome Trust Sanger Institute, Wellcome Genome Campus, Hinxton, Cambridgeshire, CB10 1SA UK; 2 Department of Medicine, University of Cambridge, Box 157, Addenbrooke’s Hospital, Hills Road, Cambridge, CB2 0QQ UK; 3 London School of Hygiene and Tropical Medicine, London, WC1E 7HT UK; 4 Cambridge University Hospitals NHS Foundation Trust, Addenbrooke’s Hospital, Hills Road, Cambridge, CB2 0QQ UK; 5 Public Health England, Clinical Microbiology and Public Health Laboratory, Cambridge University Hospitals NHS Foundation Trust, Cambridge, UK

**Keywords:** *Enterococcus faecium*, Within-host evolution, Genome sequencing

## Abstract

**Background:**

*Enterococcus faecium* is a leading cause of hospital-acquired infection, particularly in the immunocompromised. Here, we use whole genome sequencing of *E. faecium* to study within-host evolution and the transition from gut carriage to invasive disease.

**Methods:**

We isolated and sequenced 180 *E. faecium* from four immunocompromised patients who developed bloodstream infection during longitudinal surveillance of *E. faecium* in stool and their immediate environment.

**Results:**

A phylogenetic tree based on single nucleotide polymorphisms (SNPs) in the core genome of the 180 isolates demonstrated several distinct clones. This was highly concordant with the population structure inferred by Bayesian methods, which contained four main BAPS (Bayesian Analysis of Population Structure) groups. The majority of isolates from each patient resided in a single group, but all four patients also carried minority populations in stool from multiple phylogenetic groups. Bloodstream isolates from each case belonged to a single BAPS group, which differed in all four patients. Analysis of 87 isolates (56 from blood) belonging to a single BAPS group that were cultured from the same patient over 54 days identified 30 SNPs in the core genome (nine intergenic, 13 non-synonymous, eight synonymous), and 250 accessory genes that were variably present. Comparison of these genetic variants in blood isolates versus those from stool or environment did not identify any variants associated with bloodstream infection. The substitution rate for these isolates was estimated to be 128 (95% confidence interval 79.82 181.77) mutations per genome per year, more than ten times higher than previous estimates for *E. faecium*. Within-patient variation in vancomycin resistance associated with *vanA* was common and could be explained by plasmid loss, or less often by transposon loss.

**Conclusions:**

These findings demonstrate the diversity of *E. faecium* carriage by individual patients and significant within-host diversity of *E. faecium*, but do not provide evidence for adaptive genetic variation associated with invasion.

**Electronic supplementary material:**

The online version of this article (doi:10.1186/s13073-017-0507-0) contains supplementary material, which is available to authorized users.

## Background

*Enterococcus faecium* is a common gut commensal and a leading cause of nosocomial infection, particularly in the immunocompromised [1]. Bacterial characterisation using multilocus sequence typing (MLST) has demonstrated that hospital-adapted *E. faecium* are genetically distinct from commensal isolates [2] and cluster within clonal complex (CC) 17, now designated as clade A1 [3]. Isolates belonging to this clade are globally disseminated and have been linked to numerous healthcare-associated outbreaks [4–6]. Their enhanced biological fitness in healthcare settings has been associated with genetic adaptations that alter survival, virulence and antibiotic resistance [1, 3, 7]. Vancomycin-resistant *E. faecium* (VRE) is of particular concern and limits treatment options [8, 9], as recognized by its inclusion in the WHO priority pathogens list for the research and development of new antibiotics [10].

The increasing application of whole genome sequencing (WGS) to clinical settings has begun to elucidate the genomic epidemiology of *E. faecium* at national and individual hospital levels [11–13]. In addition to confirming the widespread dissemination of hospital-acquired *E. faecium*, these studies have shown that WGS can track VRE transmission during complex healthcare pathways that involve multiple wards within the same hospital [14]. Less is known about the genomic dynamics and evolution of *E. faecium* within individual patients [15]. This is important as there is growing evidence that short-term within-host evolution is higher than that estimated for long-term evolution and can result in a divergent population [16], which can manifest as variable drug resistance and virulence [17, 18]. One previous longitudinal study of *E. faecium* carriage by residents of a nursing home described the presence of lineages that varied in vancomycin resistance associated with loss of *vanA* in the same individual [19]. Here, we extend these findings through the study of four patients who developed *E. faecium* bloodstream infection while undergoing longitudinal surveillance of stool carriage, and from whom multiple colonies were sequenced from stool, blood cultures and their environment.

## Methods

### Study setting and participants

Four patients were prospectively identified on two adult haematology wards at the Cambridge University Hospitals NHS Foundation Trust (CUH) between May and Nov 2015 (two cases on each ward) who developed *E. faecium* bloodstream infection during longitudinal screening for *E. faecium* carriage in stool on admission and weekly until discharge. Blood cultures were taken by the treating doctors following protocols for sepsis investigation. Blood culture sets consisting of three bottles (standard aerobic, anaerobic and FAN, BacT/ALERT, bioMérieux, Marcy l’Etoile, France) were obtained peripherally and/or centrally. Environmental samples were also obtained from patient bedside and bathroom/toilet areas from three cases on the day of discharge using flocked swabs (FLOQSwabs, Copan Italia spa, Brescia, Italy).

### Microbiology

Blood cultures were processed by the routine diagnostic laboratory. For those positive for *E. faecium*, up to ten colonies were randomly selected from each positive blood culture set by picking colonies from all positive primary plates onto which bottles were sub-cultured. Environmental swabs were inoculated into Brain Heart Infusion broth, incubated at 37 °C in air overnight and 100 μL sub-cultured onto BBL^TM^ Enterococcosel^TM^ agar (BD, Oxford, UK) supplemented with 30 mg/L ampicillin (Sigma-Aldrich, Poole, UK) to select for ampicillin-resistant *E. faecium*), and *Brilliance* VRE (Oxoid, Basingstoke, UK) to select for vancomycin-resistant *E. faecium*. A single colony was selected from each environmental sample, with priority given to vancomycin-resistant *E. faecium* if present. Stool samples were cultured onto two solid media (Enterococcosel agar with ampicillin, and *Brilliance* VRE chromogenic agar) and into two enrichment broths (Enterococcosel (BD, Oxford, UK) and Enterococcosel with 6 mg/L vancomycin). In addition, the first stool sample for each patient was cultured using media without antibiotic selection (Slanetz-Bartley agar (Oxoid), and Enterococcosel broth). Culture media and broths were incubated at 37 °C in air for up to 48 h, with the addition of shaking for liquid culture. For broths that turned black (indicative of enterococcal growth), 100 μL was sub-cultured onto Enterococcosel agar with/without ampicillin or *Brilliance* VRE, respectively. Up to five colonies were picked from each plate for confirmation of species using matrix-assisted laser desorption/ionization time-of-flight mass spectrometry (MALDI-TOF MS; Biotyper version 3.1, Bruker Daltonics, Coventry, UK). Phenotypic antimicrobial susceptibility testing was determined using the P607 card on the Vitek 2 instrument (version 7.01, bioMérieux, Marcy l’Étoile, France). Categorization into susceptible, intermediate and resistant was done according to EUCAST clinical breakpoints (http://www.eucast.org/clinical_breakpoints/) except for high-level gentamicin and high-level streptomycin resistance, which were validated against CLSI synergy testing (500 and 1000 mg/L, respectively).

### Sequencing and population genomics analysis

DNA was extracted and DNA libraries prepared and sequenced on an Illumina HiSeq2000 with 100-cycle paired-end runs as described previously [20] to give an average depth of 85-fold. MLST sequence types (STs) were derived from Illumina read data using the script available at https://github.com/sanger-pathogens/mlst_check. A Velvet-based algorithm [21] was used to produce de novo multi-contig assemblies, which were annotated with Prokka [22], and the output used to reconstruct the pan-genome using Roary [23]. A core genome was created for each isolate using a 99% cut-off, with a default 95% identity cut-off. Scoary [24] was used with 50 re-samplings in the permutation test to find significant associations between the presence and absence of genes in the pan-genome and antibiotic susceptible/resistant and carriage/disease phenotypes. Single nucleotide polymorphisms (SNPs) were extracted from the core-genome alignment produced by Roary using the script available at https://github.com/sanger-pathogens/snp-sites. Sites with ambiguous calls were removed and the output used to construct a neighbour-joining phylogenetic tree with the ape and phangorn packages in R. The tree and associated metadata were visualised using iTOL [25] and Figtree (http://tree.bio.ed.ac.uk/software/figtree/).

### Population structure and phylogenetic Bayesian analysis

We used the core genome alignment produced by Roary and extracted the SNPs with an in-house tool, available at https://github.com/sanger-pathogens/snp-sites. We then used the SNP presence or absence as the input for Scoary to identify variants strongly associated with the source of isolation. The SNP alignment was also used to estimate the genetic population structure by hierBAPS [26], which is a nested clustering approach, with five, seven and ten values for estimated number of clusters and two iterations. After identifying four BAPS clusters that contained isolates associated with bloodstream infection (referred to here as BAPS 1, 2, 3 and 4), a Bayesian phylogenetic tree was obtained for isolates within each group so as to obtain a more accurate picture of recent evolution within each BAPS group. To achieve this, the reads were first mapped to a nominated reference sequence in each cluster based on the best assembly statistics (largest N50 value). SMALT v0.7.4 (http://www.sanger.ac.uk/science/tools/smalt-0) was then used on each cluster with the maximum and minimum insert sizes of 1000 and 50, respectively. SAMtools mpileup and BCFtools were used to detect SNPs, with the parameter values of a minimum base quality of 50 and minimum root mean squared mapping of 30 to identify SNPs. SNPs at sites in which the variant was present in < 75% of paired-end reads were excluded. Mapping to a reference genome can be inaccurate if the reference genome has a different structure based on genomic re-arrangements or other genetic variation. However, in our analysis the local reference was highly similar to the other isolates within the same BAPS group (>99% identity) and is unlikely to have a significantly different genomic architecture.

We then used Gubbins [27] with five iterations to remove high SNP density regions, which are indicative of recombination. To assess the temporal signal for each cluster, the root-to-tip distance values obtained by constructing a neighbour-joining tree was plotted against time (day) of isolation, using 10,000 bootstraps with re-sampled days to attain a distribution for R-squared values. The significance of the real R-squared was evaluated based on the distribution.

BEAST v1.7 [28] was then used on the SNP alignment to obtain the Bayesian tree. Isolates were assigned based on the sample from which each was cultured (blood culture, stool or environmental sampling), which were defined as a discrete “source” trait and then used to reconstruct the counts of state change, as detailed in [29]. BEAST was run using date of isolation and various models that included a strict molecular clock model with uniform and lognormal distribution for base frequency and a lognormal distribution for the transition of location status. Three independent MCMC chains were run for 50 million generations (sampling every 1000 generations). The convergence was confirmed if the effective sample size exceeded 200, after excluding the first 10% states as burn-in. The TreeAnnotator tool, which is part of the BEAST package, was then used to obtain confidence intervals for the node ages. The key parameters, including tree root height and clock rate, of different runs from different models were in agreement.

Using the alignment from the Bayesian analysis, the SeqTrack function in the Adegenet R package was used to construct a network for within-host isolates for each BAPS cluster. This reconstructs the most parsimonious network structure based on pairwise SNP distances and date of isolation [30, 31]. The results were visualized using functions in the igraph package in R.

The marginal likelihood values for the ancestral state for presence/absence of plasmids were estimated with the rerootingMethod function (the SYM function) in the phytools R package. The results were then displayed with the plotTree function.

### Identification of virulence factors, antibiotic resistance and plasmids

Virulence factors, resistance genes and plasmid replicons were identified using the srst2 package [32] using 90% coverage and 10% divergence cut-offs. Virulence factors were curated by extracting known and putative virulence factors for *E. faecium* from multiple sources, including the virulence factor database (http://www.mgc.ac.cn/VFs/). The list of virulence factors is available in Additional file 1. The plasmid replicon database and antibiotic resistance genes were downloaded from the srst2 package. The presence of plasmids was further evaluated by reconstructing plasmid assemblies using plasmidSPAdes [33], based on their coverage. After confirming the presence of a plasmid, the genomic context of resistance genes was confirmed by comparing the plasmid containing the gene of interest against the non-redundant nucleotide NCBI database using BLAST. Paired-end reads were then mapped to the reference genome of known previously sequenced plasmids using the same approach as above to confirm the presence of the plasmid. The sequence and the annotation files of the plasmid in BAPS2 group, reported in [14], are available in Additional file 2.

## Results

**Fig. 1:**
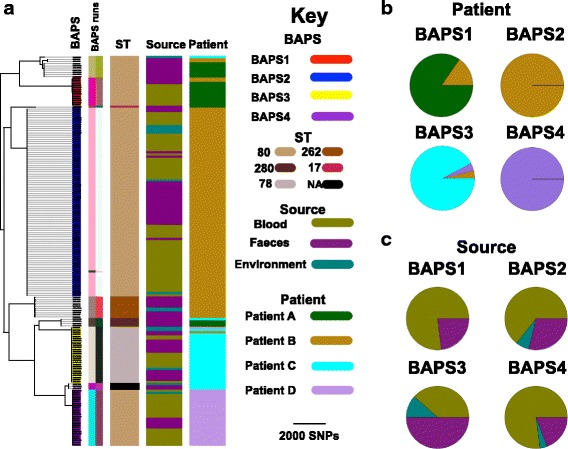
**a** Neighbour-joining tree of 180 *E. faecium* genomes used in this study. BAPS clusters 1, 2, 3 and 4 are inferred BAPS groups that all contained invasive isolates. The pie charts for BAPS groups 1, 2, 3 and 4 contain 13, 87, 26 and 26 isolates, respectively. The BAPS groups were on average 4797.97 SNPs apart. The average pairwise SNP distance for isolates within BAPS1, 2, 3 and 4 were 3.97, 2.13, 2.86 and 1.30, respectively. The BAPS run columns correspond to clustering results with five and ten values for the estimated numbers of clusters in the hierBAPS analysis. Each colour signifies one group. BAPS groups 1, 2, 3 and 4, which contained invasive isolates, were inferred with both parameter sets. The root of the tree is the midpoint of the two most distant taxa in the collection. Frequency of isolates based on patient (**b**) and source of isolation (**c**) across four BAPS groups

Our analysis was based on the whole genomes of 180 *E. faecium* isolates cultured from the blood and stool of four hospital in-patients (termed patients A–D) and the environment of three of these cases (see Additional file 3: Figure S1 for sampling timeline). To define the population diversity of the entire collection, a phylogenetic tree was constructed based on SNPs in the core genome (Fig. 1a). This demonstrated several distinct clones, which were highly concordant with the population structure inferred by hierBAPS (Fig. 1a). Four different BAPS groups (termed 1–4) contained isolates associated with bloodstream infection (Fig. 1a, c). Although the majority of isolates from each patient resided in a single BAPS group, the four patients also carried minority populations in stool that belonged to multiple BAPS groups, a diversity that was maintained over time (Additional file 3: Figure S2). Bloodstream isolates from each patient belonged to a single BAPS group that differed in each individual (Fig. 1a, b). BAPS1, BAPS2, BAPS3 and BAPS4 contained blood isolates from patient A, B, C and D, respectively. Bloodstream isolates in some patients were related to stool carriage isolates from other cases (Fig. 1a, b). Specifically, stool isolates from patient B resided in BAPS1 group that contained blood isolates from patient A. Furthermore, another stool isolate from patient B together with an environmental isolate from patient D resided in the same BAPS group as blood isolates from patient C (Fig. 1b). These findings suggest that isolates from bloodstream infections are closely linked with carriage isolates, which may be shared by different patients.

Multilocus sequence types (STs) were derived from the sequence data for the isolate collection. This revealed multiple STs, the majority of which were ST80 (*n* = 136) with the remainder being ST262 (*n* = 10), ST78 (*n* = 26) and ST280 (*n* = 4), all of which belong to the hospital-associated clade A1 (CC17), together with two isolates with novel STs (Fig. 1a). To confirm the clade assignment for the study isolates we compared these with the genomes of isolates from A1, A2 and B clade drawn from a diverse collection described previously [3], which were recovered from various ecological environments. The phylogenetic tree of isolates constructed from the core genome confirmed that our isolates resided in clade A1 (Additional file 3: Figure S3). We noted that STs had poor resolution in capturing the within-patient population diversity, with incomplete concordance between the ST tree and whole genome tree (see ST80 clades in Fig. 1a), which can be attributed to recombination in MLST loci. This indicates the superiority of WGS in understanding fine resolution of within-host population structure, as described previously [12, 34].

**Fig. 2:**
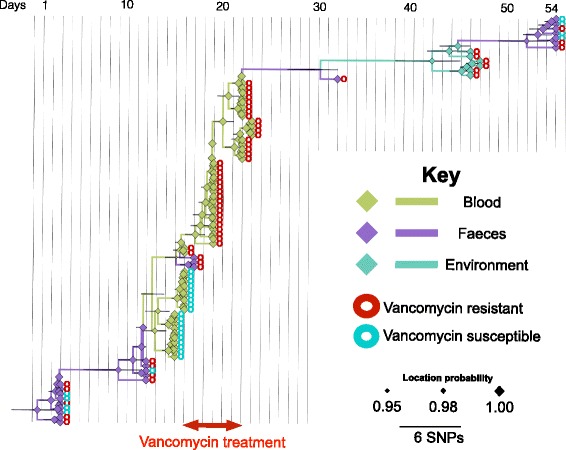
Bayesian phylogenetic tree for patient B/BAPS2 group. Branches and nodes are coloured based on the inferred origin of ancestral strains. The size of the *diamond signs* shows the posterior probability values for the inferred status. The *bars* on the nodes denote 95% confidence intervals, i.e. the credible set that contains 95% of the sampled values

For the analysis of within-host bacterial diversity, we first examined isolates from patient B who had 102 isolates (56 blood, 38 stool, eight environmental) recovered from 22 specimens (eight blood, six stool, eight environmental) over a period of 54 days, including a period of persistent bacteraemia over 9 days (Additional file 3: Figure S1). Phylogenetic analysis of the 102 isolates demonstrated that most isolates resided in BAPS2 (56 blood, 25 stool, six environmental), and were assigned to ST80, ST262 and ST78, which were each distributed across the tree (Fig. 1). The stool *E. faecium* population was assigned to BAPS1, BAPS2 and additional minor groups (Additional file 3: Figure S2). Despite the presence of multiple strain types in stool, only ST80/BAPS2 isolates from stool were closely related to isolates cultured from blood (Fig. 1a). These were present in stool before the first episode of bacteraemia, suggesting that the patient developed invasive disease with their carriage isolate (Fig. 1a; Additional file 3: Figure S2). A more detailed analysis of diversity in BAPS2 was undertaken to explore whether one or more genetic features were over-represented in invasive isolates and could be associated with the process of invasion or survival in the bloodstream. This included an analysis of mutations and the presence/absence of accessory genes. SNPs were identified in patient B/BAPS2 isolate genomes by mapping to a relevant reference genome and removing hypervariable recombinant regions (Fig. 2; Additional file 4). This identified 30 SNPs, of which nine were intergenic, 13 were non-synonymous and eight were synonymous. The non-synonymous mutations occurred in regulator proteins, ABC transporters and putative membrane proteins. A pan-genome analysis identified 250 accessory genes (~ 7% of the pan-genome) that were variable in patient B BAPS2 isolates (Additional file 5). This included genes carried by the plasmid that also carried vancomycin resistance genes (see below). The other genes were mainly insertion elements, phage-related and hypothetical proteins. Comparison of the presence of SNPs or accessory genes in blood versus other isolates, including environmental and stool isolates, did not reveal an association between the presence/absence of either an accessory gene or SNP and blood isolates (Additional file 3: Figure S4). This suggests that invasion was not linked with a particular adaptive variant.

We noted that patient B BAPS2 isolates had a strong temporal signal (R-squared value for the dataset was greater than those of 99% of replicas in the bootstrap test) and used Bayesian analysis to further explore within-host population diversity over time in this patient (Fig. 2). The substitution rate calculated for within-host *E. faecium* evolution was 128 (95% confidence interval (CI) 79.82 181.77) mutations per genome per year, which is more than ten times higher than previous estimates for *E. faecium* based on a collection of clinical isolates in which each isolate was from a different patient [14]. This discrepancy is consistent with previous suggestions that within-patient substitution rates are higher due to increased genetic drift and limited time for purifying selection to act on mildly deleterious mutations [16]. We repeated the Bayesian analysis for a subset of 20 isolates drawn from the four BAPS groups, i.e. five isolates from each BAPS group, for all four patients and found a lower substitution rate of 18.25 (95% CI 11.52 45.63) mutations per genome per year, which is closer to previous estimates [14, 35]. Due to the low number of variants within the BAPS groups, a comparison between strength of selection within and across lineages was not possible, but we identified a total of 678 variants (120 intergenic, 187 synonymous, 371 non-synonymous) that were fixed in the lineages across BAPS groups (Additional file 6). The non-synonymous variants occurred in different functional groups of genes, including membrane proteins, surface proteins, including transporters and facilitators, and regulatory genes. Whether or not these variants contain any adaptive significance needs verification from experimental functional studies.

**Fig. 3:**
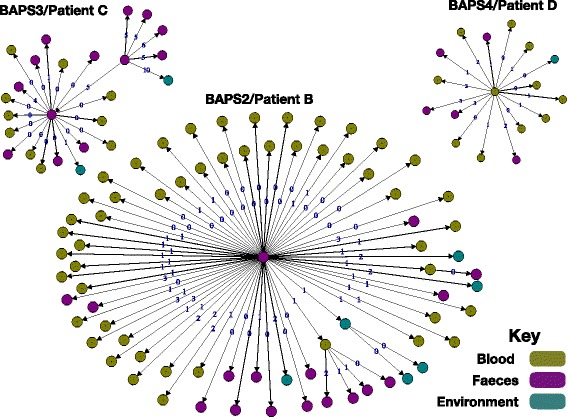
Transmission tree of BAPS2 isolates from three patients reconstructed based on genetic distance and isolate dates. The *edge numbers* denote the number of mutations. *Arrows* model potential transmissions from ancestors

The phylogenetic tree of patient B BAPS2 isolates demonstrated some mixing of blood, stool and environmental isolates but was consistent with stool isolates being ancestral to those isolated from blood as well as later isolates from stool and the environment (Fig. 2). Stool to blood transition could also be inferred from a transmission network reconstructed for BAPS2 isolates, which indicated high connectivity between stool, blood and environmental isolates (Fig. 3). In the network, carriage isolates gave rise to multiple blood isolates, some of which were closely linked with other stool and environmental isolates. This is consistent with the possible repeated translocation of *E. faecium* from gut into blood (Fig. 3). Both the transmission network and the phylogenetic tree included cases where blood isolates appeared to be ancestral to stool and environmental isolates; this is likely to be due to the presence of ancestral stool isolates that were not sampled.

**Fig. 4:**
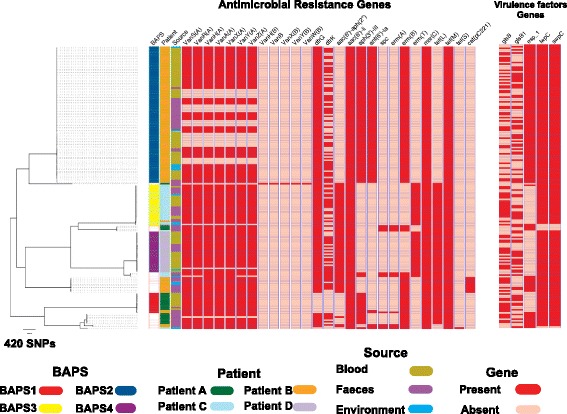
Phylogenetic distribution of genes encoding antibiotic resistance and virulence factors. Note that only virulence factors that were variably present in the population are shown, meaning that some of the well-known virulence factors, e.g. *sagA* and *atlAEfm*, present in every isolate are not shown. The sequence file for the virulence factors studied here is provided in Additional file 1. Note that the *vanB*-containing isolate was phenotypically susceptible to vancomycin due to lack of *vanR*_*B*_ and *vanS*_*B*_

**Fig. 5:**
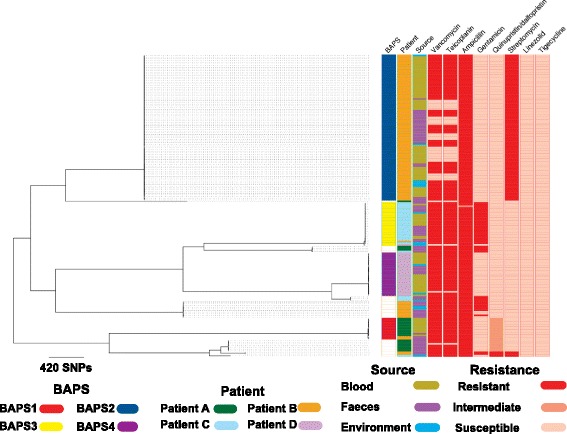
Frequency of resistant and susceptible isolates across the phylogenetic tree for antibiotics tested in this study

The presence of antibiotic resistance genes was mapped across the isolate collection from all four cases. This revealed a high degree of lineage specificity, with little variation within specific BAPS groups (Fig. 4). Only vancomycin resistance genes and *dfr* were variably present in closely related blood, stool and environmental isolates in BAPS2 (Fig. 4). Consideration of within-host isolate resistance was focused on the largest subset from patient B, who carried isolates in stool that were variably resistant to streptomycin, gentamicin and quinupristin/dalfopristin. These resided in different BAPS groups, but BAPS2 blood isolates were universally resistant to streptomycin but susceptible to gentamicin and quinupristin/dalfopristin (Fig. 5). By contrast, patient B BAPS2 isolates were variably resistant to vancomycin and teicoplanin (Fig. 5). To explore this further, we determined the genomic context of vancomycin resistance genes and the presence of transposon Tn*1546*. This demonstrated that the transposon was carried by a plasmid with the same backbone described previously in *E. faecium* isolates associated with bacteraemia at the same study hospital [14]. The *van* genes and associated plasmid were present in ancestral stool isolates but were absent in multiple strains that were recovered later (Additional file 4: Figures S5 and S6). The uncertainty about the status of the ancestral stool isolates, in terms of the presence of the plasmid and the resistance gene, suggests that the population initially contained a mixture of vancomycin susceptible and resistant isolates and that the initial bacteraemia isolates were all susceptible (Additional file 3: Figure S6). Strikingly, under vancomycin treatment, both the stool and the bloodstream population of *E. faecium* shifted to vancomycin-resistant strains. Thirty-six days after vancomycin treatment was discontinued, a susceptible population of *E. faecium* was again detectable in stool. The findings suggest a dynamic balance between vancomycin-resistant and susceptible *E. faecium* sub-populations where plasmid or *van* genes can be lost in some stool isolates in the absence of antibiotic treatment, but the resistant sub-population is maintained and selected under antibiotic pressure (Additional file 3: Figure S6).

Analysis of within-host diversity in the three other cases revealed several similarities and some differences compared with patient B. For instance, patient A isolates (*n* = 22: ten blood, 12 stool) had stool isolates that resided in three different clades (Fig. 1a). A single stool isolate in BAPS1 was genetically mixed with, and was on average 2.5 SNPs different from, the blood isolates (Additional file 3: Figure S7). Blood and stool isolates from patient A were also related to stool isolates from patient B, which were ancestral and had an average SNP distance of ~ 116 SNPs. All patient A blood isolates were vancomycin resistant due to *vanA* carried by the plasmid identified in patient B isolates (results not shown). We identified 221 accessory genes that were variably present in patient A isolates within the BAPS1 group. The stool isolate appeared to lack *fms20* encoding a cell surface protein (Additional file 5). To address whether or not the gene confers adaptive importance would require experimental verification, although we note that this gene was not differentially distributed in blood and stool isolates from other patients.

Patient C isolates (*n* = 29: ten blood, 15 stool, four environment) resided across three clades in the phylogenetic tree (Fig. 1a; Additional file 3: Figures S1 and S2). The majority (24/29) resided in BAPS3, which also contained an isolate from the environment of patient D and a stool isolate from patient B, which were ancestral to those from patient A (Fig. 1b; Additional file 3: Figure S7). In the phylogenetic tree, blood isolates formed a single clade that originated from ancestral stool isolates, and there was an indication of single or multiple introductions of isolates from stool into the environment (Additional file 4: Figure S6), the transmission network confirming that stool isolates served as sources for blood and environmental isolates (Fig. 3). Except for one environmental isolate, the other isolates in BAPS3 from patient A were vancomycin resistant and had a vancomycin resistance transposon inserted into the backbone of the same plasmid as the patient B isolates. A set of 68 accessory genes, mainly mobile genetic elements, were variably present in patient C isolates within BAPS3 (Additional file 6). In addition, eight non-synonymous, seven synonymous and four intergenic mutations and two indels were identified (Additional file 5). None of these were associated with source of isolation.

Patient D isolates (*n* = 27: 20 blood, two environmental, five stool) resided in two BAPS groups. The majority (26) clustered in BAPS4 and differed by nine SNPs (three non-synonymous, two synonymous and four intergenic), while the remaining isolate resided with isolates from patient C in BAPS3 (Fig. 1; Additional file 3: Figure S7). In contrast to other patients, *E. faecium* was not recovered from stool obtained 9 and 5 days prior to the onset of bacteraemia but blood isolates clustered with stool and environmental isolates cultured subsequently (Additional file 3: Figure S7). The *van* genes resided on a different plasmid to the other cases, the plasmid backbone contained a replicon recovered previously from an individual in New York, USA (plasmid accession ID CP018831) [36], our data showing 68% coverage of that plasmid. One blood isolate was vancomycin susceptible and lacked the whole plasmid, including the resistance gene (Additional file 3: Figure S7). We identified 147 accessory genes, some of which were plasmid related and included vancomycin resistance genes (Additional file 6).

The association between source of isolation and variations in SNPs and accessory genes was also assessed for the isolates from all four patients using Scoary (see “Methods”). However, no variant was identified that was associated with source of isolation.

We screened the collection for specific virulence genes (Additional file 7), the majority of which were present in every isolate or were lineage specific and not linked with the origin of isolates (blood versus stool) (Fig. 4). We detected variability in the presence of *gls* genes (*glsA* and *glsB*), which are involved in bile resistance and are crucial for adaptation to the intestinal environment [37], but these were not over-represented in invasive isolates compared with isolates from stool (for example, present in 48 and 52% of the blood and stool isolates in BAPS2, respectively).

## Discussion

We utilized WGS to characterize *E. faecium* associated with stool carriage and bloodstream infection in four patients over time. Each patient carried several distinct clades in their stool, but only one of these was isolated from blood in each case. In the overall collection, BAPS groups containing blood isolates also contained stool and environmental isolates, indicating that the diversity of invasive isolates is part of the broader diversity of the carriage population, and that isolates from the immediate environment were derived from carriage. A comparative genomic analysis of related isolates in stool and blood of the same patient failed to reveal adaptive mutations associated with invasive disease. This is consistent with the opportunistic nature of infection in haemato-oncology patients who have numerous risk factors for systemic infection, including the presence of intravascular and urinary catheters, neutropaenia and loss of mucosal barrier integrity. We found that sub-populations of nearly identical isolates at the core genome differed in the presence of *van* genes with evidence of loss of the *van* transposon and/or the plasmid, suggesting instability. However, the maintenance of a resistant sub-population is crucial for its survival under antibiotic pressure and can result in a rapid shift towards its dominance in both the stool and the bloodstream.

We estimated a higher substitution rate for within-host isolates than has previously been calculated for *E. faecium* from population studies. A higher substitution rate for within-patient diversity is not limited to *E. faecium* and has been reported for other major pathogens such as *Helicobacter pylori* and methicillin-resistant *Staphylococcus aureus* (MRSA), and attributed to factors such as increased drift within patients, enhanced by population compartmentalization, repeated selective sweeps and fluctuation in population size, along with limited time for removal of mildly deleterious mutations [16].

The lack of adaptive genetic changes associated with the carriage-invasive transition contrasts with the within-host analysis of other pathogens such as MRSA, where the transition of carriage to invasive status has been linked with the emergence of multiple adaptive mutations that result in truncated proteins [18]. Several explanations are possible for the lack of evidence for adaptive changes allowing invasion in *E. faecium*. Our study patients were receiving treatment for haematological malignancy and so were immunocompromised, had reduced integrity of mucosal barriers, intravenous devices and repeated exposure to antibiotics. Such circumstances are associated with colonization by drug-resistant bacteria, and translocation of bacteria from the gut into the bloodstream [38]. It is also the case that hospital-adapted *E. faecium* belonging to CC17 have acquired a genetic repertoire associated with persistence and nosocomial infection, a pre-adaption that may be sufficient to facilitate invasive disease [2, 39]. Although our study systematically studied *E. faecium* recovered from the stool and blood of patients over time, our cases were restricted to patients who were immunocompromised, and only a small number were studied. This is a potential limitation of the study, and invasive disease in people who are immunocompetent may still be associated with specific adaptive bacterial changes, which could be discovered with a larger sample size. A larger dataset and more comprehensive screening are required to determine whether the findings described here can be more widely generalized.

Our study adds to previous evidence for the carriage of numerous *E. faecium* lineages by hospitalized patients, and the value of using genome sequencing to detect this [14]. This means that picking a single bacterial colony from a sample for sequencing to determine the genetics of *E. faecium* carriage is likely to capture the dominant clade but will overlook minor lineages, which nonetheless could be associated with transmission and environmental contamination. We found evidence for considerable sharing of highly related isolates between stool and the environment of the same patient, which is consistent with environmental contamination. We also found sharing of highly related isolates between the four cases, which extends findings from a previous study in the same hospital of *E. faecium* transmission associated with *E. faecium* bloodstream infection [14].

## Conclusions

Sequencing of numerous *E. faecium* isolates from four patients over a period during which they developed bloodstream infection has indicated that opportunistic infection is not associated with bacterial adaptation for the transition from carriage to the invasive disease state.

## Additional files

Additional file 1: Sequence file (fasta) containing list of virulence factors studied here. (TXT 60 kb)

Additional file 2: The sequence and annotation files of the plasmid, containing vancomycin resistance genes, in the BAPS2 group. (ZIP 95 kb)

Additional file 3: Supplementary figures. (PDF 920 kb)

Additional file 4: List of SNPs identified within the BAPS groups for each patient. (CSV 10 kb)

Additional file 5: List of accessory genes identified within the BAPS groups for each patient. (CSV 31 kb)

Additional file 6: List of SNPs identified across lineages. (CSV 109 kb)

Additional file 7: List of virulence factors and their corresponding publication and evidence. VFDB refers to the virulence factor database. (CSV 2 kb)

Additional file 8: List of accession numbers and metadata for isolates. (CSV 56 kb)
