# Supplementary material for: Within-host evolution of Enterococcus faecium during longitudinal carriage and transition to bloodstream infection in immunocompromised patients
Source: Genome Med. 2017 Dec 27;9:119. doi: 10.1186/s13073-017-0507-0 (PMC5744393; doi:10.1186/s13073-017-0507-0)
Supplement: Supplementary file 3 — Supplementary figures. (PDF 920 kb) [file 13073_2017_507_MOESM3_ESM.pdf]

Supplementary Figures

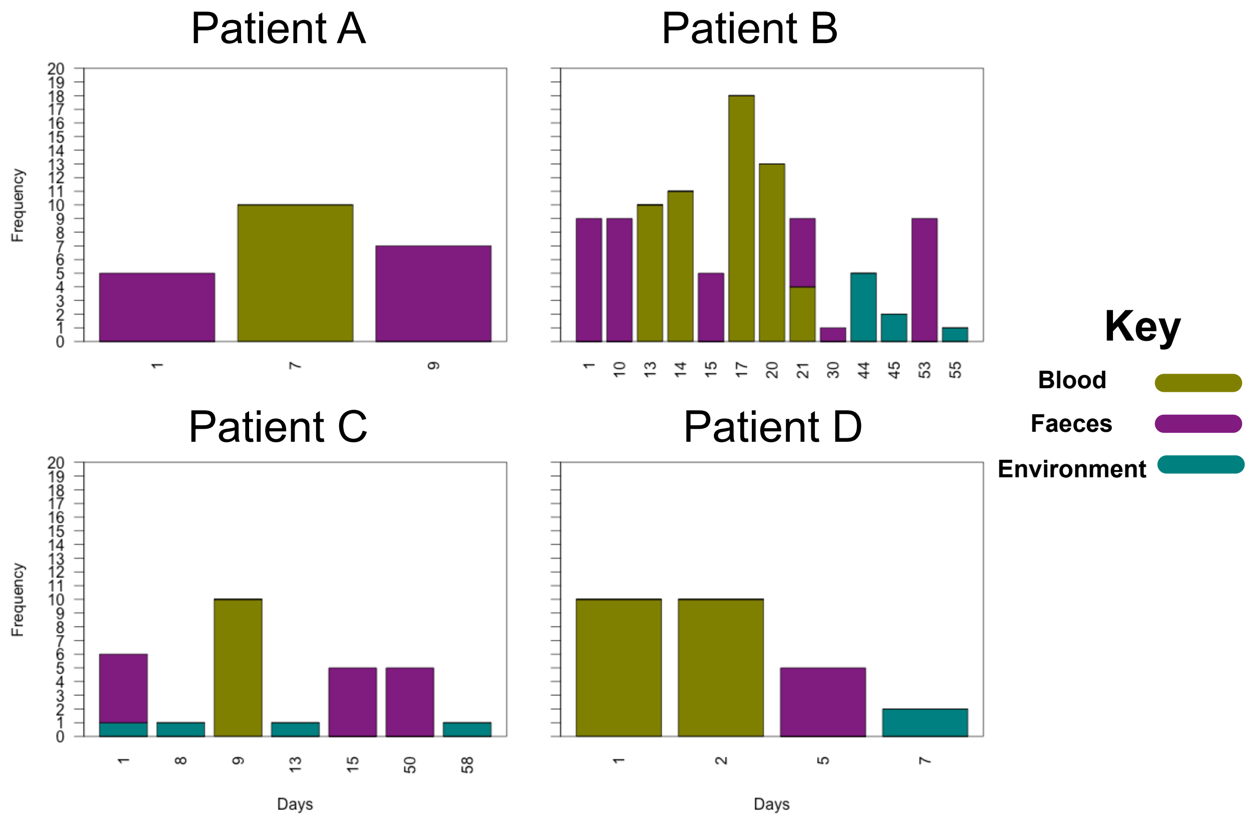

Supplemental Figure S1 Barplots show source of isolation over time for the 180 study *E. faecium* isolates from four patients.

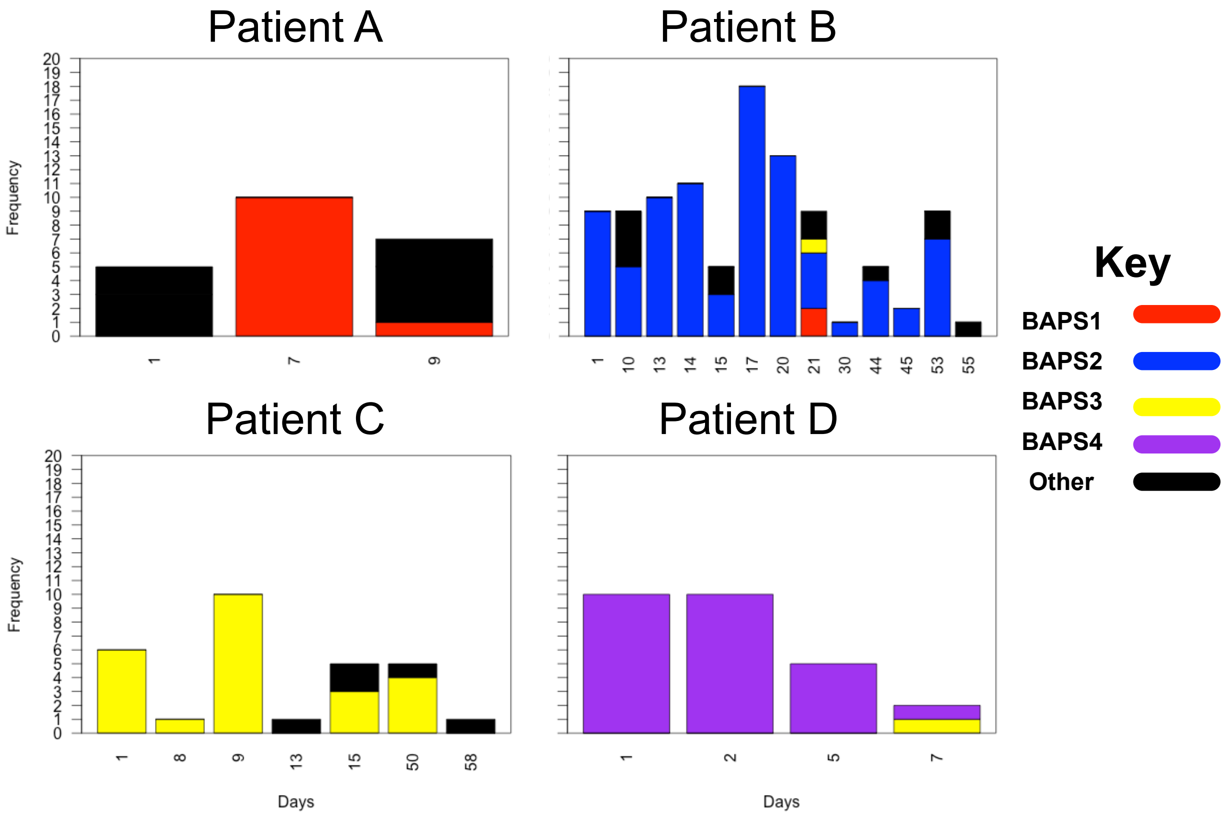

Supplemental Figure S2 Barplots showing assignment of isolates to the four BAPS groups that contained bloodstream isolates from each patient.

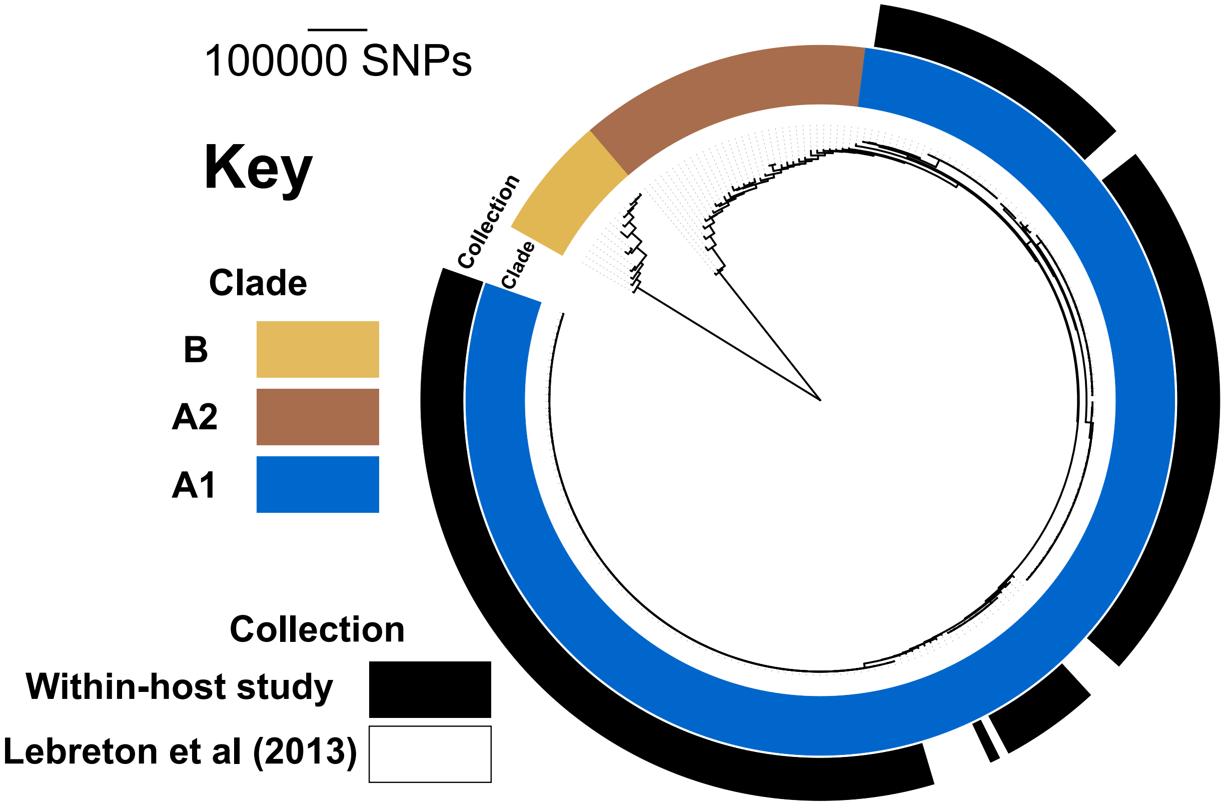

Supplemental Figure S3 Phylogenetic tree of the isolates in the study (the black outer band) and representative global isolates from Lebreton et al 2013.

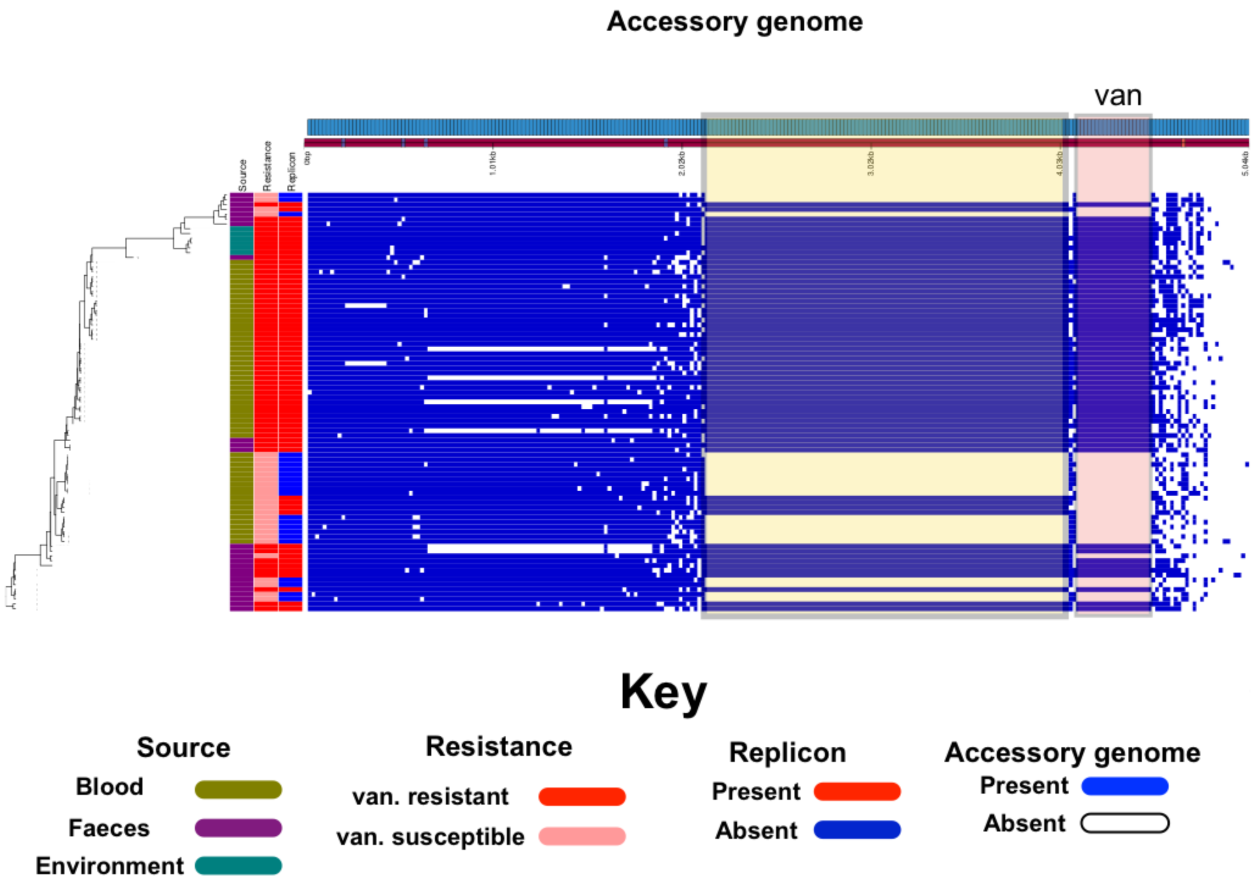

Supplemental Figure S4 Distribution of accessory genes for BAPS2 group isolates (right) from patient B, across the phylogenetic tree of the core genome (left). The yellow box shows genes located on a plasmid. The red box shows the genomic region containing vancomycin resistance genes. Genes are sorted according to their frequencies in the accessory genome. A full list of the genes and their functions is available in Supplemental Table S4.

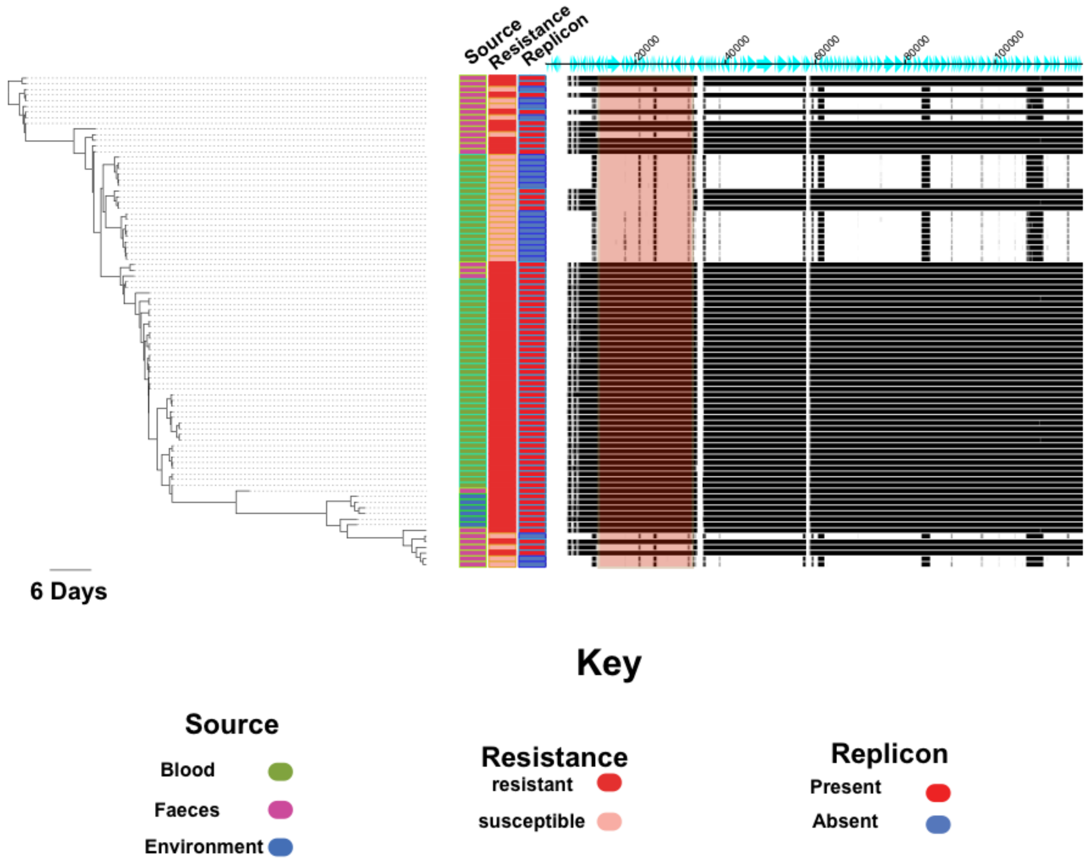

Supplemental Figure S5 Phylogenetic tree of the BAPS-2 isolates from patient B and the coverage pattern of the reference plasmid. The density of the black color corresponds to the coverage, which indicates the presence of the genomic region. The red box shows the *van* transposon genomic region in the reference plasmid and an approximately 9.5 kB downstream region flanked by IS*1216E*.

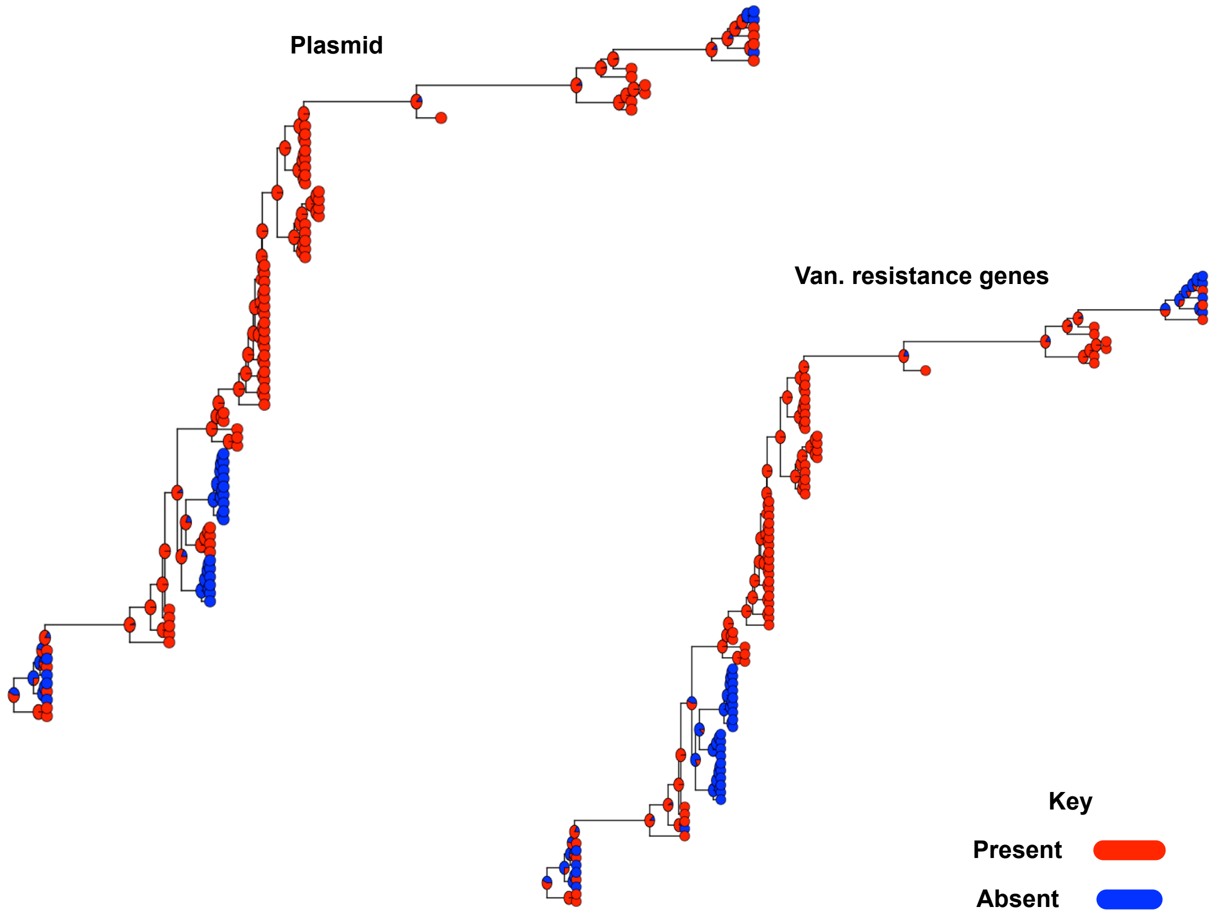

Supplemental Figure S6 Ancestral state reconstruction for the presence or absence of the vancomycin resistance cassette and the plasmid that may contain the vancomycin resistance gene. The pie charts denote the likelihood for the presence/absence of the genes/plasmid.

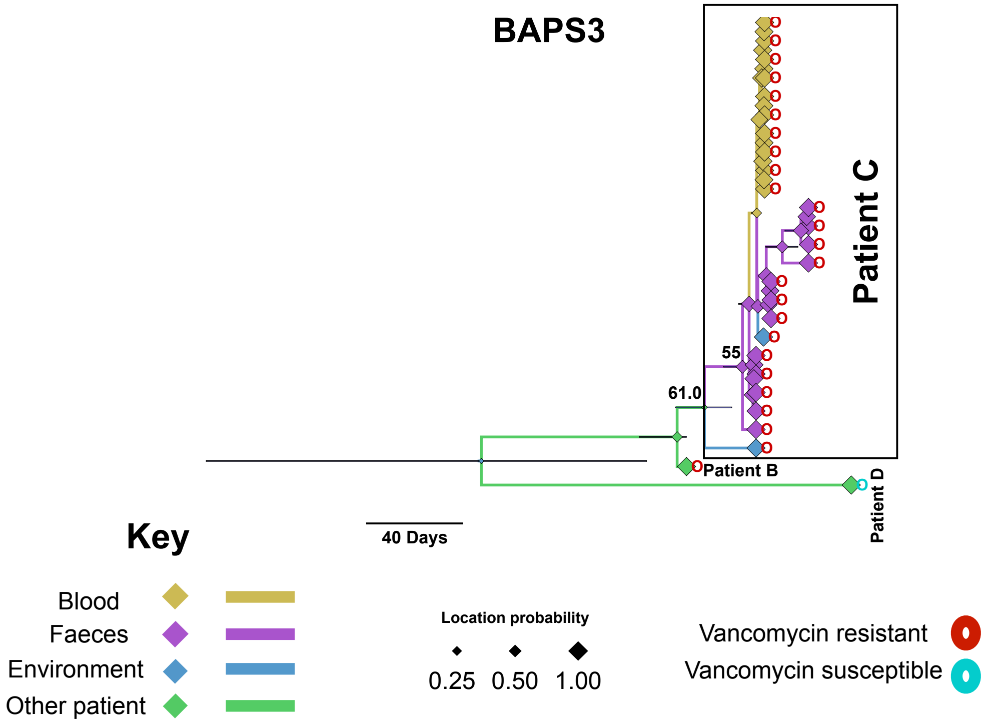
BAPS3
Patient C
55
61.0
Patient B
Patient D
40 Days
Key
Blood
Faeces
Environment
Other patient
Location probability
0.25
0.50
1.00
Vancomycin resistant
Vancomycin susceptible

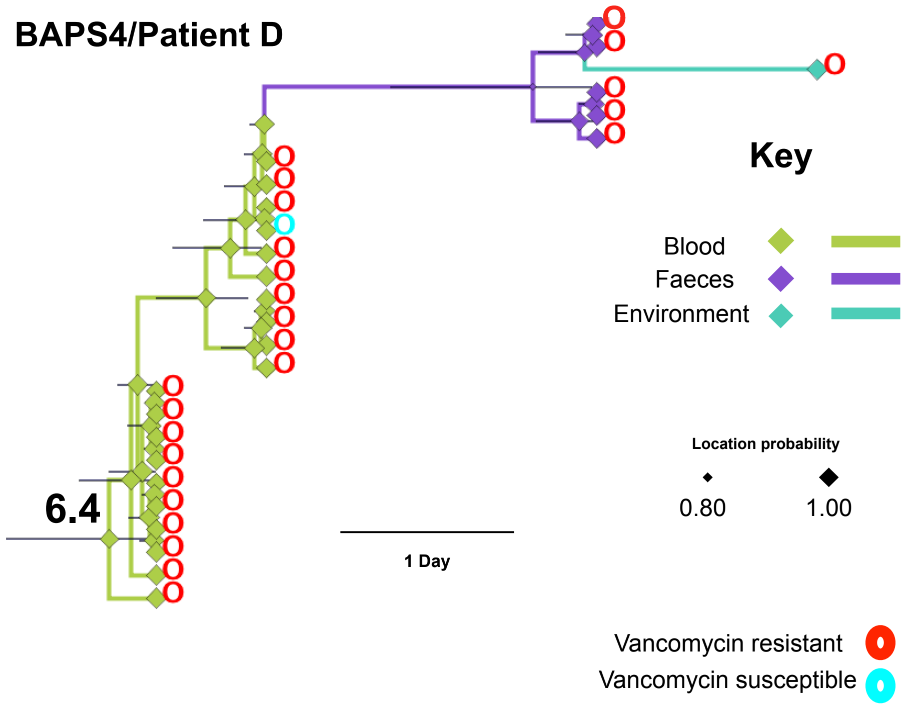
BAPS4/Patient D
Key
Blood
Faeces
Environment
6.4
1 Day
Location probability
0.80
1.00
Vancomycin resistant
Vancomycin susceptible

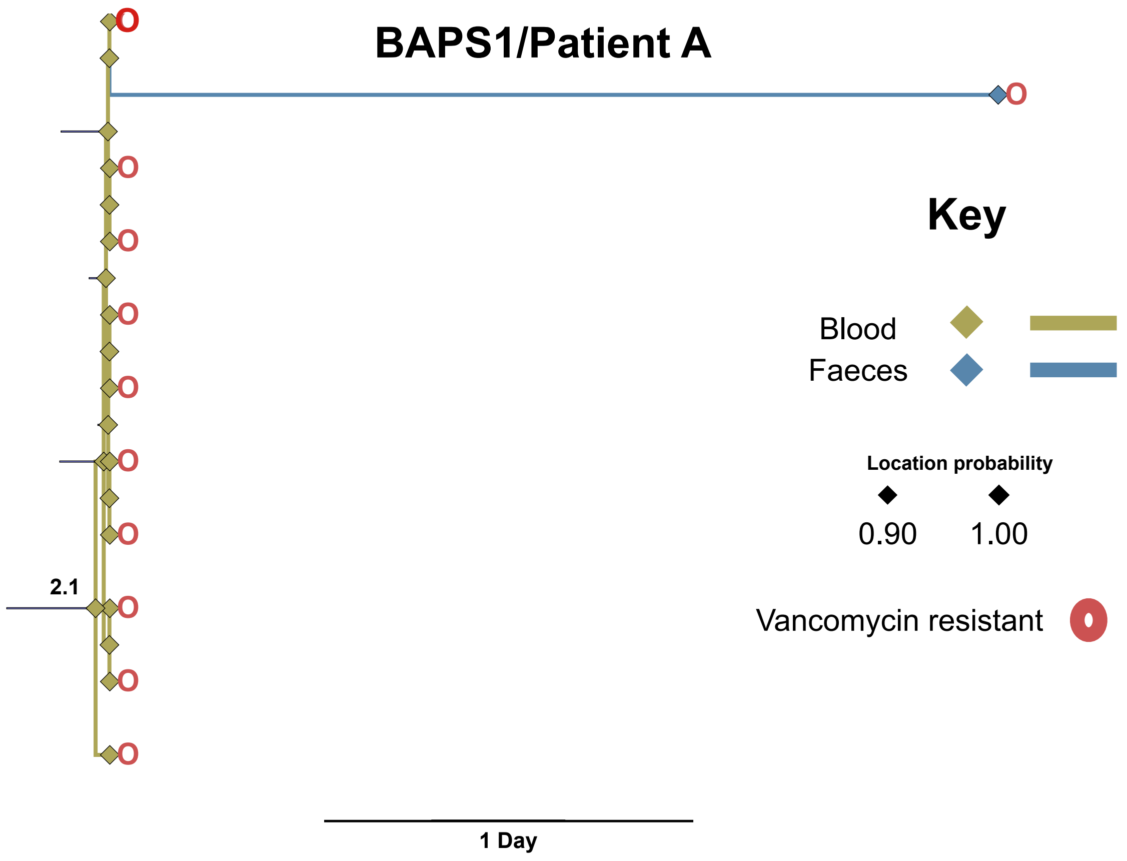

Supplemental Figure S7 Bayesian tree for the other major BAPS groups based on 30, 20 and 10 SNPs for BAPS4, 3 and 1 clusters, respectively. BAPS group 4, 3 and 1 were composed of isolates from patients D, A and C, respectively. The node numbers denote isolation days. The branch colors are the same as in Figure 2.
